# Dr. Thomas Brian Gunning

**Published:** 1889-02

**Authors:** 


### Obituary.

DIED: January 8, 1889, DR. THOMAS BRIAN GUNNING, at No. 42 Stuyvesant Place, New Brighton, Staten Island, N. Y.; age, 76 years.

Dr. Gunning was born in London, England, but came to this country at an early age and began the study of dentistry in 1840 in the office of John Bundell, one of the most famous dental surgeons of that time. His experience in this office coupled with his natural inventive genius, soon placed him in a high position in the profession. In 1861 he introduced the hard-rubber interdental splints for the treatment of fractured jaws.

In April, 1865, Dr. Gunning was called to Washington, at the suggestion of Surgeon B. F. Bache, Director of the United States Laboratory, to attend William H. Seward, then Secretary of State, who had sustained a double fracture of the jaw, by being thrown from his carriage, the case being further complicated by the attempted assassination. Dr. Gunning enabled Mr. Seward to attend a Cabinet meeting the day after a splint was applied. Dr. Gunning was one of a commission to select the medical instruments to be sent to the Paris Exposition of 1867. In the Centennial Exposition of 1876, Dr. Gunning's exhibits explained his peculiar modes of treatment completely, showing what was required for every description of fractured jaw.

In 1867 he published a pamphlet on the "Physiological Action of the Muscles Concerned in the Movement of the Lower Jaw," in 1874 a pamphlet on "The Larynx, the Source of Vocal Sounds," and in 1878 on "Hard Rubber Appliances for Congenital Cleft Palate." He retired from practice three years ago. He leaves two sons and one daughter.

